# Impact on clinical outcomes, surgical interventions, anaesthetic decisions and complication rates following implementation of the NICE obstructive sleep apnoea guidelines during preoperative screening

**DOI:** 10.1016/j.clinme.2024.100266

**Published:** 2024-11-18

**Authors:** Gabrielle Shaw, Ricki Leggatt, Paige Roberts, Amanda Peace Witton, Nicole Moll, Akshay Dwarakanath

**Affiliations:** a Department of Respiratory Medicine, Mid Yorkshire Teaching NHS Trust, Wakefield, England; b Department of Anaesthesia, Mid Yorkshire Teaching NHS Trust, Wakefield, England

**Keywords:** OSA, Preoperative screening, CPAP, Complications, NICE guidelines

## Abstract

**Introduction:**

Unidentified obstructive sleep apnoea (OSA) can lead to unexpected perioperative complications, unplanned postoperative admissions and increased length of hospital stay. NICE (National Institute for Health and Care Excellence) recommends a rapid preoperative assessment for patients undergoing elective surgery.

**Methods:**

We have evaluated the impact on implementing the NICE guidelines on clinical outcomes, surgical interventions, anaesthetic decisions and complication rates in surgical patients referred from the pre-assessment clinic prior to an elective intervention. All patients with a clinical suspicion of OSA based on a STOP-Bang score of 3 or more were referred for an overnight oximetry. Demographics, clinical outcomes and the impact on the planned surgical procedures were evaluated.

**Results:**

450 patients (Age 55 ± 14 years, male 69%, Epworth Sleepiness Scale (ESS) 7 ± 5) with a STOP-Bang score of 3 or more underwent overnight oximetry (32%; normal, 44%; mild, 15%; moderate and 9%; severe OSA). All patients with moderate and severe OSA were recommended for continuous positive airway pressure (CPAP) therapy to facilitate their surgical procedures and for long-term cardiometabolic benefits. Diagnosis of moderate/severe OSA had an impact on the surgical decision (*P* < 0.0001, odds ratio (OR) = 3.79, 95% confidence interval (CI) = 2.39–6.02). Severity of OSA affected the planned anaesthetic route (*P* < 0.0001, OR = 3.94, 95% CI = 2.21–7.05). No significant difference in day case vs non-day case, or need for unplanned admissions to critical care due to better planning pre-procedure. CPAP was initiated preoperatively in a third of patients (mean compliance 3.75 hours/day) and the overall complication rate was 11.6% in the moderate/severe OSA group vs 9.6% in the normal/mild OSA group.

**Conclusion:**

Prevalence of OSA is high in presurgical patients identified through preoperative screening. A diagnosis of moderate to severe OSA impacts surgical decision and planned anaesthetic route. Prior awareness of the diagnosis may help clinicians to identify the at-risk group. Timely CPAP initiation to facilitate surgery remains a challenge and, despite low compliance, CPAP may reduce postoperative complications. A multidisciplinary team (MDT) approach and a dedicated CPAP pathway post-diagnosis may help the clinicians and patients.

## Highlights

• Implementing the NICE OSA guidelines in preoperative patients is beneficial but poses challenges.
• Validated screening tool and diagnostics identifies a high prevalence of OSA with minimal symptoms.
• Diagnosis of moderate to severe OSA leads to surgical delays and changes in anaesthetic plans.
• A prior knowledge of OSA may lead to effective surgical triaging.
• Though CPAP therapy may play a role in preventing complications, the acceptance rate is low.
• An MDT approach and a dedicated CPAP pathway post-diagnosis may help the clinicians and patients.

## Graphical abstract

**Figure:**
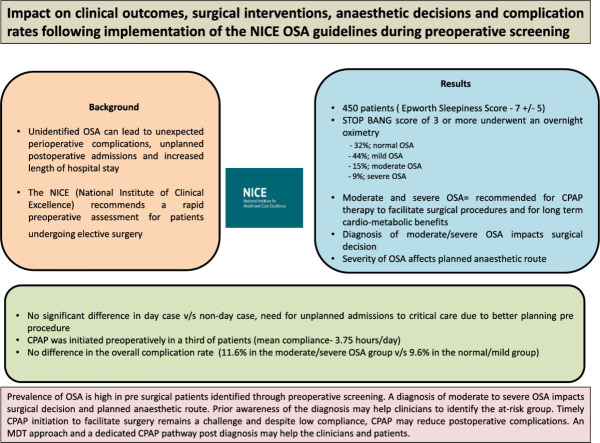

**Summary box**

**What is known?**

Unidentified obstructive sleep apnoea (OSA) can lead to unexpected perioperative complications, unplanned postoperative admissions and increased length of hospital stay.

**What is the question?**

What is the impact of implementing the NICE guidelines in preoperative OSA screening?

**What was found?**

Prevalence of OSA is high in presurgical patients identified through preoperative screening. A diagnosis of moderate to severe OSA impacts surgical decision and planned anaesthetic route. Prior awareness of the diagnosis may help clinicians to identify the at-risk group. Timely CPAP initiation to facilitate surgery remains a challenge and, despite low compliance, CPAP may reduce postoperative complications. An MDT approach and a dedicated CPAP pathway post-diagnosis may help the clinicians and patients.

**What is the implication for practice now?**

Guidelines should focus on patient risk stratification tools to reduce the clinical burden on sleep services that are already overwhelmed in the post-pandemic era. At-risk patients should be screened by a combination of subjective and objective methods and treatment optimised for the benefit of the patients and clinicians. Large multicentre randomised controlled trials are needed to address the real effect of CPAP in the surgical cohorts.

Alt-text: Unlabelled box

## Introduction

The prevalence of obstructive sleep apnoea (OSA) is increasing due to changing lifestyles, ageing population and increasing obesity incidence.^1^ OSA is often undiagnosed, and unidentified and untreated OSA is associated with high morbidity, mortality and has implications preoperatively.^2^, ^3^, ^4^ Unidentified OSA can lead to difficult intubation,^5^ unexpected perioperative complications, unplanned postoperative admissions, increased length of hospital stays and poor surgical outcomes.^6^, ^7^, ^8^, ^9^

Continuous positive airway pressure (CPAP) is considered the gold standard in the management of OSA for symptom control, improving quality of life and to reduce long-term cardiometabolic complications.^10^ Preoperative screening of patients for OSA is worthwhile, independent of any effect of CPAP upon surgical outcomes; younger and less symptomatic patients are identified earlier.^11^ Initiation of CPAP therapy during the preoperative period decreases the severity of postoperative sleep disordered breathing and improves oxygen saturation in patients with moderate to severe OSA.^12^ In a comparative cohort study of postoperative outcomes, there was an increased incidence of cardiovascular complications, primarily cardiac arrest and shock, in undiagnosed OSA but not in those diagnosed who were treated with CPAP.^5^ Furthermore, patients with OSA who were not compliant with CPAP had the highest rate of postoperative complications.^13^ Therefore perioperative CPAP initiation in patients undergoing major surgical intervention may have a role, though this needs to be confirmed in a prospective randomised controlled trial.

The recent National Institute for Health and Care Excellence (NICE) guidelines recommend a rapid sleep assessment for those with suspected OSA who are due to undergo major elective surgery.^10^ Preoperative clinical assessment of all patients with a suspicion of OSA in a dedicated sleep clinic is not a pragmatic solution. This would strain the sleep services already struggling to meet demand in the post-pandemic era.^14^ There is no study that has gauged the clinical impact of implementing the NICE guidelines in the preoperative cohort. Implementing the NICE guidelines was a clinician-driven exercise and we hypothesised that OSA prevalence would be higher in this cohort of patients, would impede the flow of various surgical interventions due to delays in diagnostic assessment, increased burden on sleep clinics and timely initiation of CPAP.

### Aims of the study

In this study we have evaluated the prevalence of OSA in patients undergoing elective surgical interventions, assessed the impact of preoperative OSA diagnosis on planned surgical interventions, anaesthetic decisions, and impact of preoperative CPAP set-up, compliance and have compared the complication rates between OSA severities.

## Method

### Preoperative OSA screening pathway (see supplementary materials)

This was developed specifically to identify the at-risk patients assessed in the dedicated pre-assessment clinic before the planned surgical intervention. All adult patients scheduled for elective surgery were included in the screening. Patients were excluded from screening if they had a known diagnosis of OSA or were on CPAP, those with a previous normal sleep diagnostic test in the previous 24 months or were scheduled for an OSA treatment surgery. Eligible patients were assessed either by an anaesthetist or a nurse prior to surgical intervention. The STOP-Bang questionnaire, a well-validated screening tool among the surgical cohorts, was used to determine the OSA risk^15^ (see supplement).

Patients who had a score of ≥3 were referred to the sleep clinic to undergo diagnostic testing using an ambulatory, overnight pulse oximeter (PULSOX-300i, Konica Minolta sensing wristwatch). The sampling rate of the watch was set to 1 second and study duration of > 4 h was considered as optimal for analysis. Patients also completed an Epworth Sleepiness Scale (ESS) prior to oximetry testing (see supplement). The oximetry was formally reported by a sleep physician and the standard threshold of 4% was used for defining significant desaturations, and the severity of OSA was determined by an oxygen desaturation index (ODI) of <5 (normal), 5–15 (mild), 16–30 (moderate) and >30 (severe).^16^ CPAP therapy was recommended in all patients with moderate to severe OSA to facilitate their surgical procedures, and for long-term cardiometabolic benefits. Additionally, any patients who reported significant sleepiness (ESS >10) were also offered a CPAP trial following a sleep clinic review. CPAP initiation and follow-up were undertaken by sleep clinical nurse specialists and patients were encouraged to use the device for at least 4 h per night.

### Patient selection

This was a single-centre retrospective observational study, and all patients who underwent an overnight oximetry following a STOP-Bang score of ≥3 over an 18-month period (November 2021 – April 2023) following the implementation of the screening pathway were included. Baseline demographics (age, gender, BMI, type of surgery, STOP-Bang score, ESS score and ODI) were recorded. The turnaround time from the time of request to reporting and reasons for delay were documented.

The patient's anaesthetic plan including the decision to delay or cancel surgery was obtained from each patient's preoperative notes. The planned level of ward stay post-surgery was also documented, with the options for this being day case procedure, inpatient, level 1 or high dependency unit (HDU). Level 1 care was defined as enhanced ward-based care with a better patient-to-nurse ratio (4:1). Postoperative notes were then used to identify whether any complications had occurred. This included anything during the surgery and up to the patient being discharged. The complications were categorised into respiratory, cardiovascular and anaesthesia related. CPAP data use was obtained from the trust's dedicated sleep database. Postoperative notes were used to determine which of these patients used their CPAP device in recovery and post-surgery.

### Statistical analysis

Statistical analysis was performed on IBM SPSS statistics (version 29). Statistical significance was set at *P* < 0.05. For ease of presentation, patients were classified into two groups; normal/mild OSA and moderate/severe OSA. Categorical data are presented as number (n), percentage (%), adjusted odds ratio (OR) and 95% confidence interval (CI). Continuous data are presented as the mean ± the standard deviation where normally distributed and presented as the median, interquartile range (IQR) and range where distribution is skewed. The Pearson chi-square test was used to compare categorical data, Fisher's exact test where one or more of the 2 × 2 contingency table contained a frequency of <5 and unpaired *t*-test for continuous data.

## Results

### Patient characteristics

450 patients with a STOP-Bang score of 3 or more underwent overnight oximetry. The patient characteristics and the various planned surgical interventions are shown in Table 1. The oximetry was completed and reported in 6 ± 4 days (mean ± SD). In less than 2% of patients the oximetry had to be repeated due to either a failed or a suboptimal study. 51% (*n* = 230) had no comorbidities, 37% (*n* = 167) had cardiovascular (heart failure, arrhythmias and hypertension) and 12% (*n* = 53) had metabolic comorbidities.

**Table 1:** Baseline demographics and the various planned surgical interventions.

Age (mean ± SD)	55 ± 14
Male; female	69% (*n* = 311); 31% (139)
Epworth Sleepiness Scale (mean ± SD)	7 ± 5
BMI (mean ± SD)	32 ± 6
Types of surgical Interventions	
Upper and lower gastrointestinal	8%
Breast	1%
ENT	8%
General surgery	14%
Gynaecology	4%
Laparoscopic interventions	4%
Orthopaedics	26%
Maxillofacial	5%
Ophthalmology	3%
Plastics	6%
Urology	20%
Vascular	1%

SD, Standard deviation, ENT, Ear nose and throat.

### Clinical outcomes and prevalence of OSA

**Fig. 1:**
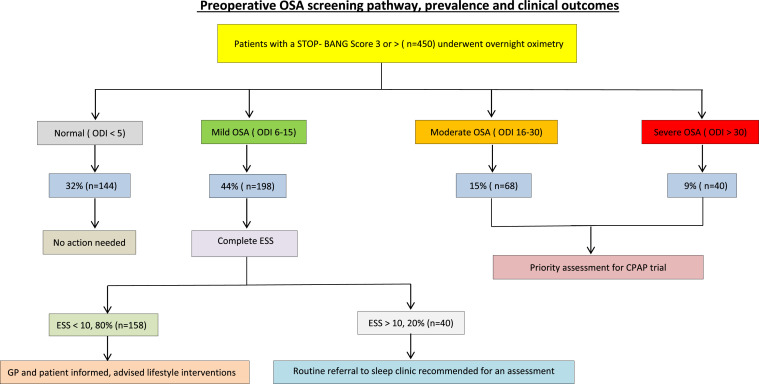
Consort diagram showing the prevalence of OSA following overnight oximetry and the clinical outcomes.

32% (*n* = 144) had a normal oximetry, 44% (*n* = 198) had mild OSA, 15% (*n* = 68) had moderate OSA and 9% (*n* = 40) had severe OSA. As STOP-Bang score increased, the proportion of normal and mild OSA decreased and moderate to severe OSA increased but the ESS remained low. This is shown in Table 2 and also in the consort diagram-1 (Fig. 1).

**Table 2:** STOP-Bang score, oximetry outcomes and ESS.

STOP-Bang Score	Normal (ODI < 5)	Mild OSA (ODI 6–15)	Moderate OSA (ODI 16–30)	Severe OSA (ODI > 30)	ESS (mean)
3 (*n* = 57)	53% (*n* = 30)	39% (*n* = 22)	7% (*n* = 4)	1% (*n* = 1)	8
4 (*n* = 83)	43% (*n* = 36)	42% (*n* = 35)	10% (*n* = 8)	5% (*n* = 4)	7
5 (*n* = 120)	35% (*n* = 42)	45% (*n* = 54)	13% (*n* = 16)	7% (*n* = 8)	7
6 (*n* = 96)	18% (*n* = 17)	50% (*n* = 48)	23% (*n* = 22)	9% (*n* = 9)	7
7 (*n* = 49)	18% (*n* = 9)	37% (*n* = 18)	27% (*n* = 13)	18% (*n* = 9)	8
8 (*n* = 15)	13% (*n* = 2)	27% (*n* = 4)	33% (*n* = 5)	27% (*n* = 4)	8

Numbers are representative where data were available. OSA, Obstructive sleep apnoea; ODI, oxygen desaturation index, ESS, Epworth Sleepiness Scale.

### Impact of OSA on surgical and anaesthetic decisions

**Fig. 2:**
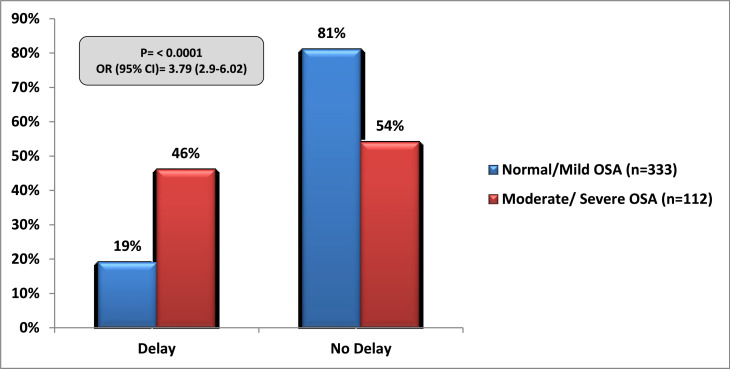
Impact of OSA diagnosis on planned surgical interventions.

**Fig. 3:**
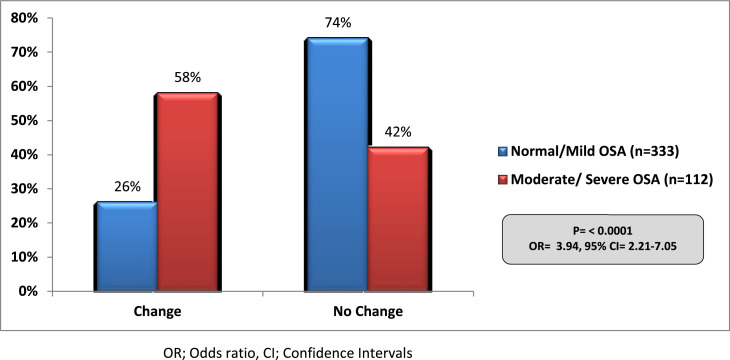
Impact of OSA diagnosis on planned anaesthetic decisions.

A preoperative diagnosis of moderate or severe OSA led to a delay in the planned surgical intervention (*P* < 0.0001, OR = 3.79; 95% CI = 2.9–6.02) and also influenced the clinicians to alter the planned route of anaesthesia as compared to those diagnosed with normal or mild OSA (*P* < 0.0001, OR = 3.94; 95% CI = 2.21–7.05 respectively). This is shown in Fig. 2, Fig. 3. There was no significant difference on day case procedures, critical care admissions or unplanned upgrade of postoperative care between normal/mild OSA and moderate/severe OSA patients. This is shown in Table 3.

**Table 3:** Impact of OSA diagnosis on day case interventions, critical care admissions and unplanned upgrade of postoperative care.

Day case	Yes	No	P-value
Normal/mild OSA	69% (*n* = 188)	31% (*n* = 83)	NS
Moderate/severe OSA	65% (*n* = 39)	35% (*n* = 21)	
Critical care admissions	**Yes**	**No**	
Normal/mild OSA	5% (*n* = 14)	95% (*n* = 257)	NS
Moderate/severe OSA	7% (*n* = 4)	93% (*n* = 56)	
Unplanned upgrade of postoperative care	**Yes**	**No**	
Normal/mild OSA	3% (*n* = 9)	97% (*n* = 262)	NS
Moderate/severe OSA	3% (*n* = 2)	97% (*n* = 58)	

### Pre-operative CPAP set up and compliance

CPAP therapy was initiated in a third of patients (*n* = 35) preoperatively following a review in the sleep clinic (led by either a clinician or a specialist nurse) and 4% had undergone a previous failed CPAP trial. The duration from the referral to clinical review for CPAP was 6 ± 5 weeks. Mean compliance of CPAP was 3.75 hours per night at the end of 6 months.

### Complication rates

Patients (*n* = 33, 26 with either a normal oximetry or mild OSA and seven with moderate to severe OSA) had complications. There was no significant difference in the overall complication rate between the normal/mild OSA and moderate/severe OSA groups (9.6% v/s 11.6%, *P* = 0.63). The complications noted were respiratory (difficult airway, prolonged apnoea, rapid desaturations, increased oxygen requirements), cardiovascular (arrhythmias, pulmonary oedema) and anaesthesia related (difficult airway and slow recovery in the postoperative period). There was no significant difference in the preoperative CPAP compliance in patients who had complications compared to those who did not (3.75 hours v/s 4.5 hours).

## Discussion

Untreated OSA is an independent risk factor for poor surgical outcomes and increasing healthcare burden. Preoperative optimisation with CPAP facilitates surgical interventions in the high-risk group and a rapid assessment is recommended by NICE. There is no study that has evaluated the impact of implementing this guideline in clinical practice and we have attempted to address this issue. This was a post-intervention study evaluating a hospital-based population who were assessed prior to an elective surgical intervention in a dedicated pre-assessment clinic.

The prevalence of OSA in our study was 69%, which is consistent with other epidemiological studies.^11^ A validated questionnaire (STOP-Bang) with a high sensitivity and a low specificity was employed to identify the at-risk patients rather than the standard ESS questionnaire. These patients are not symptomatic and generally do not have excessive sleepiness, as evidenced by a low ESS. However, a low STOP-Bang score of 3 still had a 8% chance of identifying the high risk group (moderate to severe OSA) and a high STOP BANG score of 8 had a 40% chance of identifying a low-risk group (normal or mild OSA) respectively. Objective diagnostic testing should follow questionnaire screening as there are a proportion of patients who had either moderate to severe OSA with a low score, or normal to mild OSA with a high score. Where screening in the pre-assessment clinics was carried out by nurses, they did not make decisions about referral for diagnostic testing or surgery independently of the questionnaire or without the direction of the clinicians. Preoperative screening may be a form of 'opportunistic screening' in identifying the at-risk group and early CPAP therapy may reduce cardiovascular risks even in asymptomatic or minimal symptomatic patients.^17^

A preoperative diagnosis of moderate to severe OSA has tripartite implications for patients, clinicians and for the healthcare sector. Patients are more likely to face a delay in undergoing their planned surgical interventions; clinicians are more likely to alter the care provision during and after the intervention to reduce the perioperative incidents; and there is increased burden on sleep services for providing timely clinical review and CPAP trial initiation to facilitate the planned surgery. This poses a significant challenge to sleep services, leading to longer waiting lists. On the contrary, prior awareness of a diagnosis of OSA and CPAP treatment optimisation may lead to effective triaging for day case interventions, planned critical care admissions, avoiding unnecessary upgrade during postoperative monitoring and reducing complication rates that can occur due to multifactorial reasons such as sedation, anaesthetic effects, opioid use and perioperative aggravation of other comorbidities. All these factors may lead to diminished arousal response, exacerbating the hypoxaemic effects of OSA.^18^ The planned route of anaesthesia was decided prior to patients undergoing oximetry. This was changed at the discretion of the anaesthetists based on the preserved risk and they were the final arbiter.

Timely clinical assessment in the sleep services and subsequent CPAP therapy initiation preoperatively was a challenge. Completion of this task occurred in a third of patients due to multiple reasons. These include, but were not limited to: a) cancellation of surgical intervention due to very high patient risk; b) patient's decision not to proceed with the surgical intervention; c) patients may have opted for lifestyle interventions as the first-line treatment modality, given the lack of symptoms; d) CPAP therapy or the surgical intervention performed by an external provider (neighbouring NHS trust or private healthcare) and e) the impact of industrial action by the medical workforce leading to clinic cancellations may have contributed. Despite cancellation of surgical intervention, patients with moderate/severe OSA were invited for clinical assessment and possible CPAP therapy. Due to clinical capacity constraints, these patients were seen on a routine rather than a priority basis.

Less than 2% of patients refused a trial with CPAP and they were discharged with lifestyle interventions. 4% had prior experience of a failed CPAP trial in a previous healthcare sector, but an extended trial was considered in these patients.^19^ CPAP compliance was suboptimal and this was mainly due to the fact that patients being referred from the preassessment clinics do not necessarily perceive that they have a problem with sleep disordered breathing; their main interest is the planned surgery. However, the optimal duration of CPAP needed to have a beneficial effect and the long-term outcomes in the surgical population has not been confirmed and will need a randomised controlled trial. We did not find a significant difference in the complication rate between the two groups and this may be due to preoperative awareness of OSA and the role of CPAP in controlling the sleep disordered breathing, leading to better and safer planning by the clinicians.

Our study had limitations. Firstly, this was a retrospective observational study and some data were missing and data cleansing was done before final analysis. Secondly, the patient population was exclusively those who were being assessed for an elective intervention. All acute and oncological interventions did not undergo screening and thus the true prevalence may have been underestimated. Thirdly, to ensure that preoperative patients are diagnosed as timely and cost efficiently as possible, overnight pulse oximetry was considered in place of either a limited channel sleep study or a polysomnogram. Fourthly, review in the sleep clinic for a clinical assessment and for CPAP trial required a referral from the GP, but this was not mandatory. A referral was advised for audit, coding, governance and to capture clinical activities. A copy of the oximetry report was sent to the patient's GP and they were aware that OSA had been identified as part of preoperative assessment. We think it is highly unlikely that GPs would have overruled the referral request in the interest of the planned anaesthetic safety and surgery. Fifthly, we did not include serum bicarbonate as a biomarker and also to increase the specificity of STOP-Bang screening.^20^ Finally, we did not have the long-term CPAP data at the time of analysis but this is ongoing.

In conclusion, the impact of implementing the NICE guidelines in preoperative patients is both beneficial and poses challenges. Screening with a combination of both a validated tool and diagnostic test identifies a high prevalence of OSA patients who are minimally symptomatic. A diagnosis of moderate to severe OSA leads to delays in the planned surgical interventions and changes in anaesthetic plans. A prior knowledge of OSA may lead to effective surgical triaging. Though CPAP therapy may play a role in preventing complications, the acceptance rate is low. Future directions should include designing a pragmatic pathway to facilitate the planned surgical interventions that identifies the at-risk group based on validated screening tools, cost-effective diagnostic tests, use of biomarkers, timely initiation of CPAP therapy with an aim to improve compliance and reduce the burden on sleep services nationally.

## Data availability statement

The raw/processed data required to reproduce the above findings cannot be shared at this time as the data also form part of an ongoing study.

## Ethics approval and consent to participate

Formal ethical approval was obtained from the Manchester Metropolitan University (reference number: 58795), as well as the research and development department within the Mid Yorkshire Teaching NHS Trust. No patient identifiable data was used in the study and no informed consent was obtained from the participants given that this was a retrospective observational study. The ethical committee deemed that this was not required.

## Funding

This research did not receive any specific grant from funding agencies in the public, commercial, or not-for-profit sectors.

## CRediT authorship contribution statement

**Gabrielle Shaw:** Writing – original draft, Methodology, Data curation. **Ricki Leggatt:** Formal analysis, Data curation. **Paige Roberts:** Data curation. **Amanda Peace Witton:** Data curation. **Nicole Moll:** Conceptualization. **Akshay Dwarakanath:** Writing – review & editing, Writing – original draft, Visualization, Validation, Supervision, Resources, Project administration, Methodology, Investigation, Formal analysis, Data curation, Conceptualization.

## Declaration of competing interest

The authors declare that they have no known competing financial interests or personal relationships that could have appeared to influence the work reported in this paper.

## Appendix Supplementary materials

Image, application 1

Image, application 2
