# Supplementary material for: Impact on clinical outcomes, surgical interventions, anaesthetic decisions and complication rates following implementation of the NICE obstructive sleep apnoea guidelines during preoperative screening
Source: Clin Med (Lond). 2024 Nov 18;25(1):100266. doi: 10.1016/j.clinme.2024.100266 (PMC11696840; doi:10.1016/j.clinme.2024.100266)
Supplement: Supplementary file 1 [file mmc1.docx]

**OSA screening pathway for pre-operative patients**

No need for OSA screening

Needs screening for OSA

Yes

No

STOP- BANG Score 3 or >

Complete ESS

If score 10 or <= No action needed

If score > 10, refer for sleep services

Refer to Sleep services

Severe OSA

Moderate OSA

Mild OSA

Refer for Overnight Oximetry
