# Supplementary material for: Impact on clinical outcomes, surgical interventions, anaesthetic decisions and complication rates following implementation of the NICE obstructive sleep apnoea guidelines during preoperative screening
Source: Clin Med (Lond). 2024 Nov 18;25(1):100266. doi: 10.1016/j.clinme.2024.100266 (PMC11696840; doi:10.1016/j.clinme.2024.100266)
Supplement: Supplementary file 2 [file mmc2.docx]

**Supplement**

**STOP-Bang questionnaire**

**S**noring (do you snore loudly?)

**T**iredness (do you often feel tired, fatigued, or sleepy during the daytime?)

**O**bserved Apnoea (has anyone observed that you stop breathing, or choke or gasp during your sleep?)

High Blood **P**ressure (do you have or are you being treated for high blood pressure?)

**B**MI (is your body mass index more than 35?)

**A**ge (Are you older than 50 years?)

**N**eck circumference (is your neck circumference greater than 40cm?)

**G**ender (are you male?)

*Score 1 point for each ‘yes’ response.*

**B – Epworth sleepiness scale questionnaire**

*What is your chance of dozing off in each of the following situations? 0 – never, 1 – low chance, 2- medium chance, 3 – high chance. Add your numbers up to give a total score.*

1. Sitting and reading

2. Watching TV

3. Sitting inactive in a public place

4. As a passenger in a car for an hour without a break

5. Lying down to rest in the afternoon

6. Sitting and talking to someone

7. Sitting quietly after lunch

8. In a car, sat a red traffic light
